# Role of Efflux Pumps in the *in vitro* Development of Ciprofloxacin Resistance in *Listeria monocytogenes*

**DOI:** 10.3389/fmicb.2018.02350

**Published:** 2018-09-27

**Authors:** Xiaobing Jiang, Tao Yu, Ping Xu, Xiaobo Xu, Shengdong Ji, Wujun Gao, Lei Shi

**Affiliations:** ^1^College of Life Sciences, Henan Normal University, Xinxiang, China; ^2^College of Life Science and Technology, Xinxiang University, Xinxiang, China; ^3^Institute of Food Safety and Nutrition, Jinan University, Guangzhou, China

**Keywords:** *Listeria monocytogenes*, efflux pump, Lde, ciprofloxacin, resistance

## Abstract

Efflux is a primary fluoroquinolone resistance mechanism in *Listeria monocytogenes*. In the present study, ciprofloxacin resistant strains were selected by exposure of sensitive strain to progressively increasing concentrations of ciprofloxacin and then the roles of efflux pumps Lde and MdrL in the development of resistance to ciprofloxacin were also investigated in *L. monocytogenes*. Ciprofloxacin sensitive strain of *L. monocytogenes* exhibited reduced susceptibility to this antibiotic after induction. Cross-resistance to ethidium bromide (EtBr) was observed in ciprofloxacin-induced strains. However, cross-resistance to benzalkonium chloride (BC) did not occur in this study. Compared to the wild-type strain HL06, the expression levels of *lde* were increased in four ciprofloxacin-induced strains. The single-gene deletion mutants of *lde* and *mdrL* from the ciprofloxacin-induced resistant strain HL06CIP4 were constructed. However, decreased minimum inhibitory concentration (MIC) of ciprofloxacin was observed only in HL06CIP4Δ*lde* compared to that of the parental strain HL06CIP4. Ciprofloxacin uptake appeared to be obviously increased in HL06CIP4Δ*lde* in relative to HL06CIP4. These evidences suggested that efflux pump Lde is involved in ciprofloxacin resistance in *L. monocytogenes* HL06CIP4. The deletion of *lexA* had no effect on the expression levels of *lde* in HL06CIP4 in the absence or presence of ciprofloxacin, indicating that LexA was not involved in the regulation of efflux pump Lde in *L. monocytogenes*.

## Introduction

*Listeria monocytogenes*, a Gram-positive, facultative intracellular foodborne pathogen, is widely distributed in the environment. It is capable of causing severe human infections with a high fatality rate, primarily in immunocompromised individuals, the elderly, pregnant women, as well as in neonates (Gandhi and Chikindas, 2007; Drevets and Bronze, 2008). Foodborne transmission is recognized as the main route of acquisition of the listerial infections. It has been documented that more than 99% of human listeriosis results from consumption of contaminated food (Scallan et al., 2011; Manuel et al., 2015).

Earlier studies showed that *L. monocytogenes* was susceptible to most clinically relevant antimicrobials except for intrinsic resistance to cephalosporins and fosfomycin (Hadorn et al., 1993; Hof, 2003; Krawczyk-Balska and Markiewicz, 2016). However, increasing numbers of strains resistant to one or more antimicrobials have been reported during the recent years (Lungu et al., 2011; Chenal-Francisque et al., 2014; Yu and Jiang, 2014). Although quinolones are not recommended as the options for listeriosis, they can promote the emergence of resistant *L. monocytogenes* strains indirectly due to their extensive use for the treatment of multiple infections (Godreuil et al., 2003).

In general, resistance to quinolone in Gram-positive bacteria results from (i) alterations in the quinolone-resistance determining regions (QRDRs) of the intracellular targets of quinolones, DNA gyrase encoded by *gyrA* and *gyrB*, and topoisomerase IV encoded by *parC* and *parE*; or (ii) decreased accumulation of drugs inside the bacteria via efflux pump systems; or (iii) Qnr-like pentapeptide repeat proteins (Rodríguez-Martínez et al., 2008; Redgrave et al., 2014; Hooper and Jacoby, 2016). Previous studies have found that efflux pumps seem to be a major mechanism for quinolone resistance in *L. monocytogenes* (Godreuil et al., 2003; Jiang et al., 2012). Until now, two efflux pumps which are associated with quinolone resistance have been described in *L. monocytogenes* (Godreuil et al., 2003; Guérin et al., 2014). Efflux pump Lde, encoded by the *lde* gene, belongs to the major facilitator superfamily (MFS). Besides fluoroquinolone resistance, Lde is also found to be involved in resistance to acridine orange and ethidium bromide (EtBr). Another fluroquinolone efflux pump, termed FepA, is the member of the multidrug and toxic compound extrusion (MATE) family.

Previously, Godreuil et al. (2003) inactivated the efflux pump gene *lde* by insertion in *L. monocytogenes* fluoquinolone resistant strain CLIP21369 (serotype of 1/2b) and the mutant showed three- to four-fold more sensitivity than its parental strain, suggesting that this efflux pump could be responsible for fluoquinolone resistance. In our previous work, we observed that *lde* was significantly overexpressed in ciprofloxacin resistant strains of *L. monocytogenes* exposed to sublethal concentrations of ciprofloxacin (Jiang et al., 2012). However, limited information is available on the role of Lde in the induced resistant mutants obtained upon exposure of *L. monocytogenes* to fluoroquinolones.

It has been found that active drug efflux in Gram-positive bacteria is mainly associated with MFS-type pumps (Li and Nikaido, 2004; Andersen et al., 2015). In *L. monocytogenes*, another MFS-pump, MdrL, has also been reported most commonly (Mata et al., 2000; Romanova et al., 2006; Tamburro et al., 2015). But, it is still unclear whether MdrL is involved in the induced resistance to fluoroquinolones in *L. monocytogenes*. Therefore, the aims of this study were to select ciprofloxacin resistant strains by exposure of sensitive strain to progressively increasing concentrations of ciprofloxacin and investigate the roles of efflux pumps Lde and MdrL in the development of resistance to ciprofloxacin in *L. monocytogenes*.

## Materials and Methods

### Bacterial Strains, Plasmids, and Growth Condition

*Listeria monocytogenes* strains, the plasmids, and *Escherichia coli* strains used for cloning experiments are presented in **Table 1**. *L. monocytogenes* HL06 with serotype 1/2c was isolated from raw pork meat in April 2008in Henan province (Yu and Jiang, 2014). This strain was sensitive to ciprofloxacin with minimum inhibitory concentration (MIC) of 0.5 μg/ml (Yu and Jiang, 2014). *L. monocytogenes* strains were grown at 37°C on brain heart infusion (BHI) agar (Becton Dickinson, Sparks, MD, United States) or in BHI broth, and *E. coli* strains were grown at 37°C on Luria–Bertani (LB) agar (Becton Dickinson) or in LB broth. Appropriate antibiotics (Sigma–Aldrich, St. Louis, MO, United States) were added to agars and broths when needed. Erythromycin was added to BHI at a concentration of 5 μg/ml and ampicillin was added to LB at a concentration of 100 μg/ml. Primers for amplification of QRDRs, for RT-qPCR, and for construction of deletion mutant and complemented strains (**Table 2**) were designed using Primer Premier 5.0 (Premier Biosoft, Palo Alto, CA, United States).

**Table 1:** Bacterial strains and plasmids used in this study.

Strain or plasmid	Genotype	Reference or source
**Strains**		
*L. monocytogenes* strains		
HL06	Wild-type strain; serotype 1/2c	Yu and Jiang, 2014
HL06CIP1	Ciprofloxacin-induced derivative of HL06	This study
HL06CIP2	Ciprofloxacin-induced derivative of HL06	This study
HL06CIP3	Ciprofloxacin-induced derivative of HL06	This study
HL06CIP4	Ciprofloxacin-induced derivative of HL06	This study
HL06CIP4Δ*lde*	HL06CIP4 with deletion of *lde*	This study
HL06CIP4Δ*mdrL*	HL06CIP4 with deletion of *mdrL*	This study
HL06CIP4Δ*lexA*	HL06CIP4 with deletion of *lexA*	This study
CHL06CIP4Δ*lde*	Complemented strain of HL06CIP4Δ*lde*	This study
HL06CIP4Δ*lde*pERL3	HL06CIP4Δ*lde* containing pERL3	This study
*E. coli* strains		
DH5α	Chemical competent strain	Biomed, Beijing, China
DH10β	Chemical competent strain	Biomed, Beijing, China
**Plasmids**		
pMAD	Cloning shuttle integration vector with a thermosensitive origin of replication for low-GC-content Gram-positive bacteria, Amp^R^ and Ery^R^	Arnaud et al., 2004
pMAD-Δ*lde*	pMAD containing homologous region up- and down-stream of HL06CIP4 *lde*	This study
pMAD-Δ*mdrL*	pMAD containing homologous region up- and down-stream of HL06CIP4 *mdrL*	This study
pMAD-Δ*lexA*	pMAD containing homologous region up- and down-stream of HL06CIP4 *lexA*	This study
pERL3	Plasmid capable of replication in *L. monocytogenes*, Ery^R^	Sibelius et al., 1996
p*lde*	pERL3 containing 1,519bp of upstream nucleotides, coding sequence, and downstream terminator sequence of *lde*	This study

**Table 2:** Primers used in this study.

Gene	Primer	Sequence (5′-3′)	Reference
**Primers for amplification of QRDRs**			
*gyrA*	GyrA-F	CGTGGAACCTCGTCGTAAA	This study
	GyrA-R	CCATAATCATCCCAGCAGTT	
*gyrB*	GyrB-F	AAGCGCGCGCGTGAAGT	Godreuil et al., 2003
	GyrB-R	CGAGATTTAGAAACGTC	
*parC*	ParC-F	AGGCGAAAGACATTTGAGT	This study
	ParC-R	TACGGTCAGTTTCATCACG	
*parE*	ParE-F	GGAAAATTAACGCCAGC	Godreuil et al., 2003
	ParE-R	TCGGTCATGATAACTAC	
**Primers for RT-qPCR**			
*lde*	RT*lde*-F	GCGATGATTTTGATGGGA	This study
	RT*lde*-R	ACCGCTGCCGTTGATAGT	
*mdrL*	RT*mdrL*-F	TAAAGTGAAAGAACCGAAGA	This study
	RT*mdrL*-R	CAAACATAATCCCCAAGC	
16S rRNA	RT16S-F	GGGAGGCAGCAGTAGGGA	This study
	RT16S-R	CCGTCAAGGGACAAGCAG	
**Primers for mutant strain construction**			
Upstream of *lde*	*lde*-1	*GC*GTCGACTTTGGCACAGCATTAGGAT (*Sal*I)	This study
	*lde*-2	*GATAGAAGAATCTAGGTGGATTT*TCTAATACAATTACCAGGAATAGGT	
Downstream of *lde*	*lde*-3	*ACCTATTCCTGGTAATTGTATTAGA*AAATCCACCTAGATTCTTCTATC	
	*lde*-4	*CG*ACGCGTGACGATGGCTTGGTTCTG (*Mlu*I)	
Upstream of *mdrL*	*mdrL*-1	*CG*GGATCCGTCCCTTGGTTCTGGCAT (*BamH*I)	This study
	*mdrL*-2	*GTTGTAAGGTAAAATGTGCTGG*AATACAACTACACTTCCCTTTCC	
Downstream of *mdrL*	*mdrL*-3	*GGAAAGGGAAGTGTAGTTGTATT*CCAGCACATTTTACCTTACAAC	
	*mdrL*-4	*CG*GAATTCTCCAATCATAAAGTTTCGTCAG (*EcoR*I)	
Upstream of *lexA*	*lexA*-1	*CG*GGATCCGGACCTAAAATAACACCCAT (*BamH*I)	This study
	*lexA*-2	*CCATGAAAATATCTAAACGC*GGGCTTTATAGAGATATTCG	
Downstream of *lexA*	*lexA*-3	*CGAATATCTCTATAAAGCCC*GCGTTTAGATATTTTCATGG	
	*lexA*-4	*CG*ACGCGTTGTTTTACTTTATGCGGAGT (*Mlu*I)	
**Primers for sequencing of the deletion area**			
*lde*	*lde*-5	TCCGTTTCCGCAACATAG	This study
	*lde*-6	GCACATTAGCCAATACCC	
*mdrL*	*mdrL*-5	TGTAAAGCAGCAGGAGTG	This study
	*mdrL*-6	AAACGACGCTAATAACCAT	
*lexA*	*lexA*-5	TCATTTGACTAAAAGGAAG	This study
	*lexA*-6	TTGAAGGTAAAGGGCTAA	
**Primers for complementation**			
*lde*	*lde*-7	*CC*GAGCTCATCGTGAACTTAATGGTTGG (*Sac*I)	This study
	*lde*-8	*CG*GGATCCATCCTCATATAACTCAAGCG (*BamH*I)	

*Restriction sites are underlined. Overlapping areas are marked with italics.*

### Antimicrobial Susceptibility Testing

Minimum inhibitory concentrations for antimicrobial agents against *L. monocytogenes* strains were determined by the broth microdilution method in accordance with the recommendations of the Clinical and Laboratory Standards Institute (CLSI, 2011). Ciprofloxacin, ampicillin, erythromycin, kanamycin, chloramphenicol, and tetracycline were purchased from Sigma–Aldrich, EtBr solution (10 mg/ml) from Takara Bio Inc. (Tokyo, Japan), and benzalkonium chloride (BC) from Aladdin Biochemical Technology Co., Ltd. (Shanghai, China). Briefly, bacterial suspensions adjusted to a turbidity equivalent to that of a 0.5 McFarland standard were further diluted 1:20 in sterilized saline solution (0.9%) and 10 μl of suspensions were transferred to the well containing 100 μl of cation-adjusted Mueller–Hinton broth (CAMHB; Becton Dickinson) plus 2.5% lysed horse blood (Oxoid, Basingstoke, United Kingdom) with different concentrations of drugs. After inoculation, the final test concentration of bacteria in each well was approximately 10^4^–10^5^ CFU/ml. Then the plates were incubated at 37°C for 24 h. The MIC values for each drug were visually evaluated as the lowest concentration at which no growth was observed. *Staphylococcus aureus* ATCC 29213 and *E. coli* ATCC 25922 were used as control strains Clinical and Laboratory Standards Institute (CLSI, 2011). Each of the tests was repeated on three separate occasions.

### Selection of Ciprofloxacin-Induced Resistant *L. monocytogenes*

Selection of ciprofloxacin-induced resistant strains was performed as previously described (Sun et al., 2011). Briefly, HL06 was grown in BHI broth for 16 h at 37°C with shaking. One hundred microliters of the inoculum was spread on BHI agar plates with increasing concentrations of ciprofloxacin (0.5, 1, 2, and 4 μg/ml) and incubated for 24 to 48 h at 37°C. A single colony was picked up from every plate and inoculated into 5 ml of BHI broth containing the same concentration of ciprofloxacin as that in the plate. The culture was incubated for 24 h at 37°C with shaking and then was spread on BHI agar plate supplemented with an increased concentration of ciprofloxacin. The selection procedure was repeated for strains which had high MIC of ciprofloxacin. Selected ciprofloxacin-induced resistant strains were passed 10 times in BHI without ciprofloxacin. HL06 and its mutant exhibited the same MICs of ciprofloxacin in CAMHB and BHI broth (data not shown).

### PCR Amplification and DNA Sequencing of the QRDRs

Genomic DNA was extracted from *L. monocytogenes* using a TIANamp Bacteria DNA Kit (Tiangen Biotech, Beijing, China). The QRDRs of *gyrA*, *gyrB*, *parC*, and *parE* were amplified by PCR using the primers in **Table 2**. The reaction mixture (50 μl) contained 2.5 U of *Taq* Polymerase (Takara), 1 × *Taq* buffer (Takara), 25 pmol of each primer, and 200 μM dNTP (Takara). Amplification conditions were as follows: DNA was denatured at 94°C for 4 min, followed by 30 cycles of denaturation at 94°C for 30 s, annealing at 50°C for 30 s and extension at 72°C for 30 s, followed by a final extension at 72°C for 7 min. The purified PCR products were sequenced at commercial company (Sangon Biological Engineering Technology & Services Co., Ltd., Shanghai, China).The deduced amino acid sequences were aligned using the Geneious (version 8.0.5) Clustal W multiple alignment tool.

### Efflux Pump Inhibition Test by Using Reserpine

To assess the contribution of efflux pump activity in *L. monocytogenes*, MICs of ciprofloxacin, EtBr, and BC were examined in the presence or absence of the inhibitor reserpine (final concentration, 20 μg/ml; Sigma–Aldrich). The plant alkaloid reserpine has been recognized as an effective inhibitor of efflux pumps, especially MFS family in Gram-positive microorganisms (Wang et al., 2016). Experiments were repeated on three separate occasions. Control cells were grown in the presence of reserpine without ciprofloxacin. This was performed to confirm that reserpine did not have an inhibitory effect on cell growth.

### RT-qPCR

Relative expression levels of *lde* and *mdrL* genes were assessed by RT-qPCR. Single colony of each strain was inoculated into 5 ml of BHI broth and incubated overnight (16 h) at 37°C. The cultures were diluted in 5 ml of fresh BHI broth (1:100) and incubated to logarithmic growth phase (OD_600_ of 0.6) at 37°C. Total RNA was harvested from 2 ml of prepared culture using RNAprep pure Cell/Bacteria kit (Tiangen) according to the manufacturer’s instruction. Two hundred nanograms of total RNA were retro-transcribed using TIANScript RT kit (Tiangen) in a final volume of 20 μl. The PCR mix consisted of 1 × SuperReal PreMix Plus (Tiangen), 100 nm of each primer, and 1 μl of cDNA in a final volume of 20 μl. Amplification was performed in the LightCycler 96 real-Time PCR system (Roche, Basel, Switzerland). The PCR program was 95°C for 5 min; 40 cycles of 95°C for 30 s, 53°C for 30 s, and 72°C for 20 s. As a final step, melting curve analysis was performed between 65 and 95°C. 16S rRNA was used as an internal control for normalization in each sample. Relative transcription levels were quantified using the 2^−ΔΔCT^ method and shown as relative fold changes (Livak and Schmittgen, 2001). All experiments were performed in triplicate independently.

### Construction of Gene Deletion Mutants

Gene deletion was performed by homologous recombination strategy, using the temperature-sensitive pMAD shuttle vector (Arnaud et al., 2004). An insert containing homologous arms up- and down-stream of the target gene was obtained by the splicing by overlap extension (SOE) PCR (Warrens et al., 1997). In order to build the vector pMADΔ*lde*, pMADΔ*mdrL*, and pMADΔ*lexA*, the inserts and pMAD were digested using appropriate restriction enzymes (Takara) and ligated into pMAD by using T4 ligase (Takara). The recombinant plasmid was transformed into chemically competent *E. coli* DH5α cells (Biomed, Beijing, China). After confirmation by sequencing, the recombinant vector was electroporated into the competent *L. monocytogenes* HL06CIP4 cells using a Gene Pulser Xcell electroporation system (Bio-Rad Laboratories, Hercules, CA, United States) operating at 1.8 kV, 25 μF, and 200 Ω. Transformants were selected on BHI agar plates containing erythromycin (5 μg/ml; Sigma–Aldrich). Single-crossover mutant was selected at 39°C with erythromycin to promote chromosomal integration and double-crossover mutant at 39°C without antibiotic to enable plasmid curing. The deletions were confirmed by PCR and sequencing.

### Complementation of *lde* Deletion Mutant

To complement *L. monocytogenes* HL06CIP4Δ*lde*, the complete *lde* open reading frame (ORF) along with its promoter was amplified from genomic DNA. After digestion with *Sac*I and *BamH*I (Takara), the PCR product was cloned into pERL3, a plasmid capable of replication in *L. monocytogenes* (Sibelius et al., 1996) and then was transformed into chemically competent *E. coli* DH10β cells (Biomed). Plasmid DNA was extracted by using TIANpure Mini Plasmid Kit (Tiangen). After confirmation by sequencing, the correct recombinant plasmid was electroporated into the *L. monocytogenes* HL06CIP4Δ*lde* strain. Transformants were selected on BHI plates with erythromycin (5 μg/ml) and the presence of *lde* was confirmed by PCR using primers *lde*-7 and *lde*-8. The complemented strain was designated as CHL06CIP4Δ*lde*. To construct the vector control strain HL06CIP4Δ*lde*pERL3, pERL3 was electroporated into the *L. monocytogenes* HL06CIP4Δ*lde* strain. Transformants were selected on BHI plates with erythromycin (5 μg/ml) and extracting the plasmid was performed to confirm the presence of pERL3.

### Accumulation of Ciprofloxacin

Ciprofloxacin uptake was assayed according to the method described previously (Giraud et al., 2000). Single colony of each strain was inoculated into 5 ml of BHI broth and grown overnight (16 h) at 37°C. The overnight cultures were diluted in 300 ml of fresh BHI broth (1:100) and incubated to OD_600_ of 0.6. Cells were harvested by centrifugation at 4000 rpm for 15 min, washed in 50 mM sodium phosphate buffer (PB buffer, pH 7.0), and resuspended in PB buffer. After addition of ciprofloxacin (final concentration of 10 μg/ml), 500 μl of samples were removed at different time intervals. Ten minutes after addition of ciprofloxacin, efflux pump inhibitor reserpine was added to the reaction mixture (final concentration, 20 μg/ml). The samples were immediately mixed with 500 μl of ice-cold PB buffer, and centrifuged at 12,000 rpm for 5 min. The pellets were washed by ice-cold PB buffer and resuspended in 1 ml of 0.1 M glycine hydrochloride (Aladdin) at room temperature overnight. The samples were then centrifuged at 12,000 rpm for 10 min. The fluorescence of the supernatant was measured with Infinite 200 microplate readers (Tecan Group Ltd., Männedorf, Switzerland) at excitation and emission wavelengths of 276 and 452 nm, respectively. The concentration of ciprofloxacin in the supernatant was calculated by comparison with a standard curve of ciprofloxacin in 0.1 M glycine hydrochloride. The results were presented as nanograms of ciprofloxacin incorporated per milligram (dry weight) of bacteria. The experiments were performed three times.

### Accumulation and Efflux of EtBr

The accumulation and efflux of EtBr were carried out as previously described (Couto et al., 2008; Viveiros et al., 2008; Paixão et al., 2009). A LightCycler 96 instrument (Roche, Basel, Switzerland) was applied to obtain the fluorescence of EtBr with the excitation and emission wavelengths of 533 and 572 nm respectively. For the accumulation assay, *L. monocytogenes* were grown in BHI broth to an OD_600_ of 0.6, centrifuged, and washed twice in PBS (Huankai). Then the suspension was adjusted to an OD_600_ of 0.3 using PBS. The cultures were incubated with 8 μg/ml EtBr and 20 μg/ml reserpine at 25°C for a 60 min period. Arbitrary units of fluorescence emitted by EtBr were recorded at the end of each cycle of 60 s. For the efflux assay, *L. monocytogenes* were loaded with EtBr under conditions that favor accumulation (25°C and presence of reserpine). When the maximum level of EtBr accumulation was reached within 60 min, the bacteria were centrifuged and the broth was replaced by: (i) PBS with glucose (Aladdin); (ii) PBS with glucose and reserpine; and (iii) PBS containing reserpine (control of minimum efflux). Glucose was used to provide an energy source for efflux of EtBr (Viveiros et al., 2008; Paixão et al., 2009). The assay was performed at 37°C, and the fluorescence of EtBr was measured at the end of every cycle of 60 s, for a 15 min period. The efflux of EtBr is presented in terms of relative fluorescence, which is obtained from the comparison between the fluorescence value observed at each point and the control of minimum efflux. Each assay was performed in triplicate.

### Growth Curve Analysis

Growth curve analysis of HL06CIP4 and HL06CIP4Δ*lde* were carried out as previously described (Markkula et al., 2012). Five colonies of each strain were individually inoculated into 5 ml of BHI broth and incubated overnight at 37°C. The cultures were diluted in fresh BHI broth (1:100) supplemented with BC (2 μg/ml) and 300 μl of each suspension was transferred to 100-well honeycomb plate (Pöntinen et al., 2015). The strains were grown in a Bioscreen C microbiology reader (Growth Curves, Helsinki, Finland) at 37°C and the OD_600_ was measured at 15-min intervals. The lag-phase duration, mean maximum growth rate, and maximum optical density of each strain were obtained using the DMFit program (ComBase; Computational Microbiology Research Group, Institute of Food Research, Colney, Norwich, United Kingdom), based on the models of Baranyi and Roberts. Correspondence between the OD_600_ values and viable cell numbers for HL06CIP4 and HL06CIP4Δ*lde* was examined by plate counts in the early logarithmic, late logarithmic, and early stationary growth phases. The statistical significances of differences between lag-phase duration, mean maximum growth rate, and mean maximum optical density of HL06CIP4Δ*lde* and those of HL06CIP4 were tested using two-tailed Student’s *t*-test (Microsoft Excel 2010).

## Results

### Cross-Resistance to EtBr in Ciprofloxacin-Induced *L. monocytogenes*

During stepwise selection with ciprofloxacin, four mutants (HL06CIP1, HL06CIP2, HL06CIP3, and HL06CIP4) exhibiting decreased susceptibility to ciprofloxacin were obtained. MICs of several antimicrobial agents against HL06 and derived mutants selected by ciprofloxacin are presented in **Table 3**. Four mutants showed 2- to 16-fold increases in MICs of ciprofloxacin. All the mutants also exhibited reduced susceptibility to EtBr, with two- to four-fold increases in MICs. All the mutants induced by ciprofloxacin exhibited no changes in their susceptibility to BC, ampicillin, erythromycin, kanamycin, chloramphenicol, and tetracycline.

**Table 3:** MICs of several antimicrobial agents against *L. monocytogenes* strains.

Strain	MIC (μg/ml)										
	CIP	CIP + RES	AMP	ERY	KAN	CHL	TET	EtBr	EtBr + RES	BC	BC + RES
HL06	0.5	ND	1	0.125	2	4	0.5	25	ND	6	ND
HL06CIP1	1	1	1	0.125	2	4	0.5	50	25	6	2
HL06CIP2	2	1	1	0.125	2	4	0.5	50	25	6	2
HL06CIP3	2	1	1	0.125	2	4	0.5	50	25	6	2
HL06CIP4	8	2	1	0.125	2	4	0.5	200	50	6	2
HL06CIP4Δ*lde*	4	2	1	0.125	2	4	0.5	100	50	6	2
HL06CIP4Δ*mdrL*	8	2	1	0.125	2	4	0.5	200	50	6	2
HL06CIP4Δ*lexA*	8	2	1	0.125	2	4	0.5	200	50	6	2
CHL06CIP4Δ*lde*	8	ND	1	0.125	2	4	0.5	200	ND	6	ND
HL06CIP4Δ*lde*-pERL3	4	ND	1	0.125	2	4	0.5	100	ND	6	ND

*CIP, ciprofloxacin; RES, reserpine; AMP, ampicillin; ERY, erythromycin; KAN, kanamycin; CHL, chloramphenicol; TET, tetracycline; BC, benzalkonium chloride; ND, not determined.*

### Efflux Pump Is Responsible for the Development of Ciprofloxacin Resistance in *L. monocytogenes*

**FIGURE 1:**
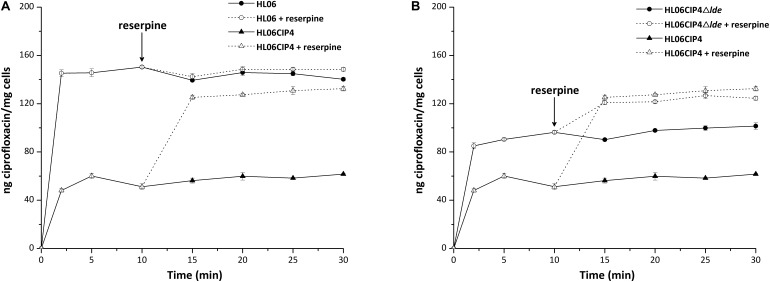
Accumulation of ciprofloxacin (final concentration of 10 μg/ml) by **(A)** HL06 and HL06CIP4 and **(B)** HL06CIP4 and HL06CIP4Δ*lde*. Reserpine (final concentration of 20 μg/ml) was added at the time indicated by the arrow. Data shown are the mean of three experiments. Error bars show the standard deviation.

No differences in QRDRs of *gyrA*, *gyrB*, *parC*, and *parE* were observed in the four ciprofloxacin-induced mutants. Among these mutants, three strains had decreases and one had no change in MICs of ciprofloxacin after being exposed to reserpine (**Table 3**). Time course ciprofloxacin uptake experiments were performed with HL06 and HL06CIP4 (**Figure 1A**). The amounts of ciprofloxacin accumulated in HL06CIP4 appeared to be lower than that in HL06 in the absence of reserpine. At steady state, reached within 2 min following addition of ciprofloxacin, HL06CIP4 accumulated about three-fold less ciprofloxacin than the wild-type strain HL06. After reserpine was added, no obvious increase in accumulation of ciprofloxacin was observed in HL06, while the drug accumulation in HL06CIP4 increased dramatically.

### Efflux Pump Lde Contributes to the Development of Resistance to Ciprofloxacin in *L. monocytogenes*

**FIGURE 2:**
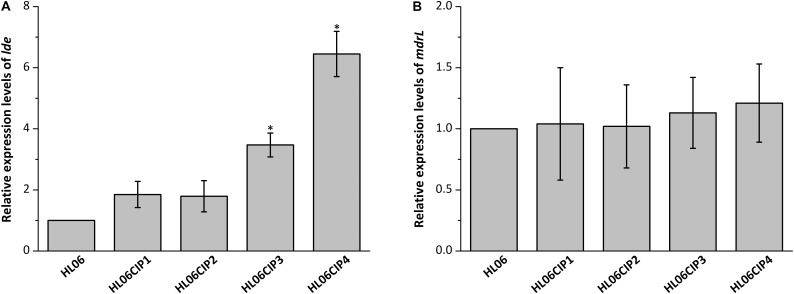
**(A)** Relative expression levels of *lde* in HL06 and four ciprofloxacin-induced strains in BHI broth. **(B)** Relative expression levels of *mdrL* in HL06 and four ciprofloxacin-induced strains in BHI broth. Results are presented as fold changes relative to the expression levels of *lde* or *mdrL* in HL06 grown in BHI. Error bars represent the standard deviation of triplicate experiments (*n* = 3). The asterisk indicates a value statistically different from that of HL06 grown in BHI, with a *P*-value < 0.05.

Results from RT-qPCR showed that the mRNA levels of *lde* in four induced mutants increased 1.85-, 1.79-, 3.47-, and 6.45-fold, respectively, compared to that in wild-type HL06 (**Figure 2A**). However, no significant difference was observed in the mRNA levels of *mdrL* between HL06 and its induced mutants (**Figure 2B**).

In order to clarify the role of Lde and MdrL in the development of ciprofloxacin resistance, the deletion mutants of *lde* and *mdrL* derived from HL06CIP4 were constructed. MICs of several compounds against the deletion mutant strains and complemented strains are presented in **Table 3**. The mutant HL06CIP4Δ*lde* showed two-fold decrease in the MICs of ciprofloxacin compared to the parental strain HL06CIP4. On the other hand, complementation of the Δ*lde* deletion strain restored the ciprofloxacin MICs of the mutant strain to the parental level. The vector control HL06CIP4Δ*lde*pERL3 had the same MICs for ciprofloxacin as the corresponding parental mutant strain. No changes in the MICs of all antimicrobial agents tested in our study were observed between the deletion mutant HL06CIP4Δ*mdrL* and the parental strain HL06CIP4.

Time course ciprofloxacin uptake experiments were also performed with HL06CIP4Δ*lde* (**Figure 1B**). In the absence of reserpine, the deletion mutant HL06CIP4Δ*lde* accumulated approximately two-fold more ciprofloxacin than the parental strain HL06CIP4 at steady state. Addition of reserpine, as expected, induced a very rapid and significant increase in cell-associated ciprofloxacin in HL06CIP4 and HL06CIP4Δ*lde*. Reserpine increased the concentration of ciprofloxacin accumulated by HL06CIP4 to 125.20 ± 1.90, and the *lde* knockout mutant to 120.83 ± 1.55 at steady state following addition of reserpine. As a consequence of adding reserpine, the accumulation of ciprofloxacin was nearly identical in HL06CIP4 and HL06CIP4Δ*lde*.

### EtBr Is the Substrate for Efflux Pump Lde

**FIGURE 3:**
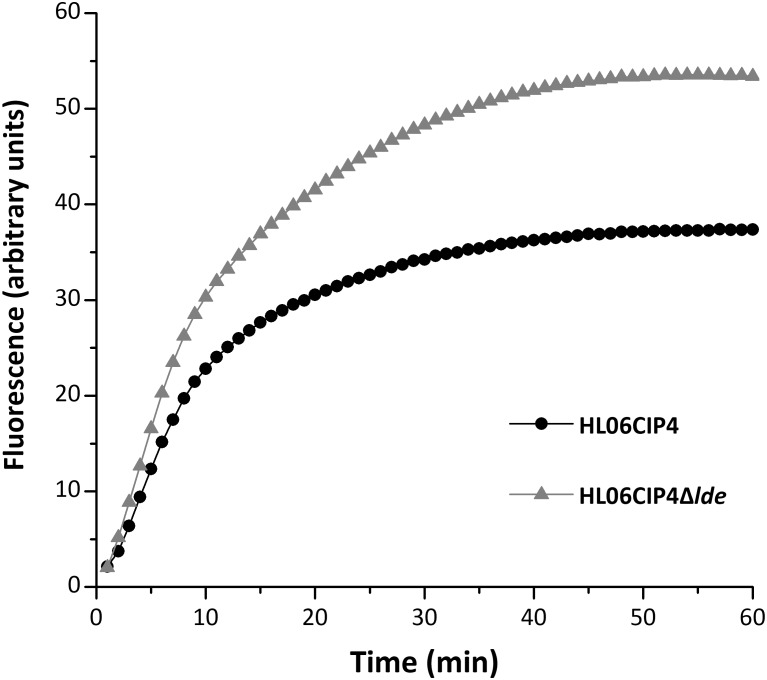
Accumulation of EtBr by HL06CIP4 and HL06CIP4Δ*lde*.

**FIGURE 4:**
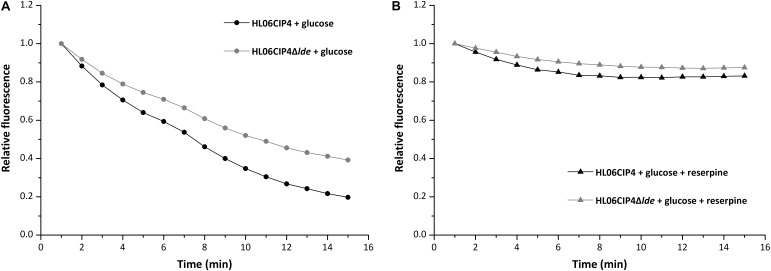
Evaluation of EtBr efflux activity for HL06CIP4 and HL06CIP4Δ*lde* under different conditions: **(A)** in the presence of glucose (final concentration of 0.4%) without reserpine and **(B)** in the presence of glucose (final concentration of 0.4%) and reserpine (final concentration of 20 μg/ml).

The mutant HL06CIP4Δ*lde* showed two-fold decrease in the MICs of EtBr compared to the parental strain HL06CIP4. Accumulation and efflux of EtBr in HL06CIP4 and HL06CIP4Δ*lde* was also determined. Our results showed that the deletion mutant HL06CIP4Δ*lde* accumulated approximately 1.5 times more EtBr relative to the parent strain HL06CIP4 (**Figure 3**). In order to investigate the efflux activity in HL06CIP4 and HL06CIP4Δ*lde*, glucose was used to provide an energy source for the extrusion of EtBr. Efflux of EtBr from HL06CIP4 and HL06CIP4Δ*lde* took place after the addition of glucose; however, the efflux activity of HL06CIP4 was about two times as much as that of HL06CIP4Δ*lde* under the same conditions (**Figure 4A**). The presence of reserpine with medium containing glucose obviously inhibited the efflux of EtBr in both strains (**Figure 4B**).

### Efflux Pump Lde Is Not Involved in Resistance to BC

**FIGURE 5:**
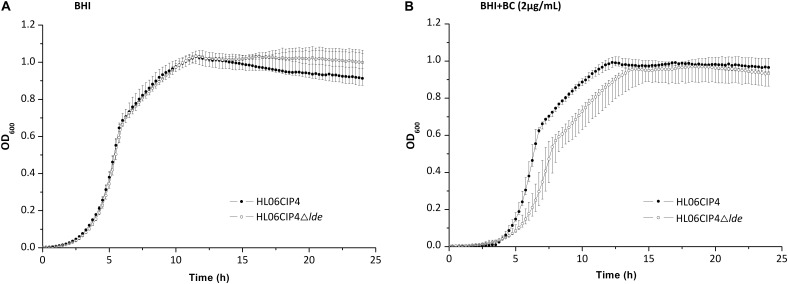
**(A)** Growth curves for *L. monocytogenes* HL06CIP4 and HL06CIP4Δ*lde* in BHI broth. **(B)** Growth curves for *L. monocytogenes* HL06CIP4 and HL06CIP4Δ*lde* in BHI broth with 2 μg/ml of BC.

The disinfectant BC presented consistent MICs that were not affected by the absence of *lde*. The growth of the deletion mutant HL06CIP4Δ*lde* was similar to that of the parent strain HL06CIP4 in BHI medium (**Figure 5A** and **Table 4**). The mutant HL06CIP4Δ*lde* showed a slightly impaired growth compared to HL06CIP4 (**Figure 5B**). However, no statistically significant differences (*P* > 0.05) were observed in lag-phase duration, maximum growth rate, and maximum optical density between HL06CIP4Δ*lde* and HL06CIP4 (**Table 4**).

**Table 4:** Average lag phase durations, mean maximum growth rates, and mean maximum optical densities of *L. monocytogenes* HL06CIP4 and its mutant HL06CIP4Δ*lde* in BHI broth with and without BC.

Growth parameter^a^	Medium	Strain	
		HL06CIP4	HL06CIP4Δ*lde*
Lag-phase duration (h)	BHI	3.050 ± 0.406	2.905 ± 0.301
	BHI + BC	4.030 ± 0.225	4.389 ± 0.135
Mean maximum growth rate ± SD (OD_600_ units/h)	BHI	0.209 ± 0.035	0.184 ± 0.014
	BHI + BC	0.180 ± 0.018	0.141 ± 0.008
Mean maximum optical density ± SD (OD_600_ units)	BHI	0.988 ± 0.048	1.016 ± 0.011
	BHI + BC	0.977 ± 0.010	0.938 ± 0.035

*^a^The statistical significances of differences between the growth parameters of HL06CIP4Δlde and those of HL06CIP4 were tested using two-tailed t-test (Microsoft Excel 2010).*

### Efflux Pump Lde Is Not Regulated by LexA

**FIGURE 6:**
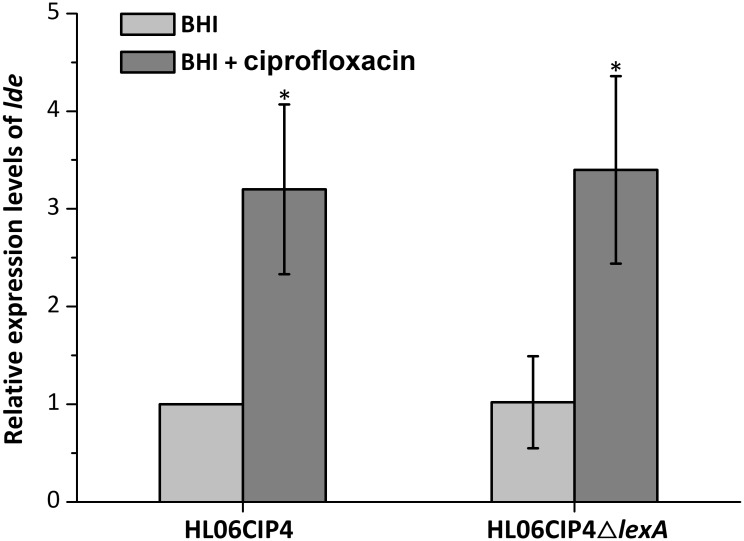
Relative expression levels of *lde* in HL06CIP4 and HL06CIP4Δ*lexA* grown in BHI with or without ciprofloxacin. Results are presented as fold changes relative to the expression level of *lde* in HL06CIP4 grown in BHI in the absence of BC. Error bars represent the standard deviation of triplicate experiments (*n* = 3). The asterisk indicates a value statistically different from that of HL06CIP4 grown in BHI, with a *P*-value < 0.05.

The relative expression levels of *lde* in HL06CIP4 and HL06CIP4Δ*lexA* are presented in **Figure 6**. In the presence of ciprofloxacin, *lde* was upregulated in HL06CIP4 (3.2-fold; *P* < 0.001) compared to the expression level without antibiotic. However, no obvious differences in the expression levels of *lde* were observed between HL06CIP4 and HL06CIP4Δ*lexA* with or without ciprofloxacin, indicating that the regulation of *lde* is independent of LexA.

## Discussion

In this study, four ciprofloxacin-induced mutants form HL06 showed increased MICs of ciprofloxacin. All the mutants also exhibited decreased susceptibility to EtBr, which was consistent with the previous study (Rakic-Martinez et al., 2011). This suggests a linkage between ciprofloxacin and EtBr resistance in *L. monocytogenes*. It was reported that *L. monocytogenes* strains induced by ciprofloxacin also displayed increased resistance to BC (Rakic-Martinez et al., 2011). However, no changes in BC MICs were observed in four ciprofloxacin-induced strains in our study. Given that a very limited number of strains were investigated in our study and the previous study, it was difficult to draw the conclusion that adaption to ciprofloxacin could or could not lead to resistance to BC. There was the possibility that different strains exhibited different resistance phenotype after ciprofloxacin induction.

Mutation in QRDRs was not detected in any of the mutants induced by ciprofloxacin, indicating that efflux pump could play a major role in the development of ciprofloxacin resistance in *L. monocytogenes*. The presence of reserpine resulted in reduced MICs of ciprofloxacin in three induced strains, supporting our hypothesis mentioned above. Unexpectedly, one strain (HL06CIP1) showed a consistent MIC for ciprofloxacin after the addition of reserpine. This observation could be explained in two ways. First, resistance to ciprofloxacin in HL06CIP1 could be due to efflux pumps that are not inhibited by reserpine. On the other hand, it was possible that resistance to ciprofloxacin in HL06CIP1 was due to other unknown mechanisms rather than mutation in QRDRs or efflux activity.

In our study, accumulation of ciprofloxacin in bacterial cells was also tested in the wild-type strain HL06 and its mutant HL06CIP4. HL06 accumulated much more ciprofloxacin than that in HL06CIP4 in the absence of reserpine. After reserpine was added, no obvious increase in accumulation of ciprofloxacin was observed in HL06, while the drug accumulation in HL06CIP4 increased dramatically. Our data indicated that HL06CIP4 had higher efflux activity for ciprofloxacin than that of HL06 and the amount of ciprofloxacin in HL06CIP4 increased remarkably in the presence of reserpine was likely due to the inhibition of efflux activity by reserpine. These results provided further evidence for our speculation that efflux pump was the major mechanism for the development of resistance to ciprofloxacin in *L. monocytogenes*. Previous studies also reported that the active efflux is associated with ciprofloxacin in *L. monocytogenes* (Komora et al., 2017).

Up to now, several MFS-type efflux pumps which are responsible for fluoroquinolone resistance have been described in Gram-positive bacteria, such as Bmr from *Bacillus subtilis* (Neyfakh, 1992), NorA from *Staphylococcus aureus* (Neyfakh et al., 1993), PmrA from *Streptococcus pneumoniae* (Gill et al., 1999), and EmeA from *Enterococcus faecalis* (Lee et al., 2003). In *L. monocytogenes*, Lde and MdrL are the most commonly investigated MFS-type efflux pumps. Lde is, according to predictions, a membrane protein containing 12 transmembrane segments with its N- and C-termini located with the cytoplasm (Godreuil et al., 2003). Lde consists of 402 amino acids, which displayed 44% identity with PmrA of *S. pneumoniae* (Godreuil et al., 2003). Previous studies have demonstrated that the presence of *lde* is ubiquitous in strains of *L. monocytogenes* examined, irrespective of their susceptibilities to quinolones, indicating that resistance is likely due to high expression level of *lde* (Godreuil et al., 2003; Romanova et al., 2006). Coincidentally, our previous study found the overexpression of *lde* in *L. monocytogenes* ciprofloxacin resistant strains when exposed to this drug for a period of time, providing further evidence that efflux pump Lde could be involved in resistance to ciprofloxacin in *L. monocytogenes* (Jiang et al., 2012). Efflux pump MdrL could be involved in resistance to antimicrobial agents such as macrolides and cefotaxime, heavy metals, and EtBr (Mata et al., 2000). However, the role of Lde and MdrL in the development resistance to ciprofloxacin was still unclear in *L. monocytogenes*.

In our study, expression of *lde* and *mdrL* in HL06 and its ciprofloxacin-induced strains were determined. Compared to the wild-type strain HL06, the expression levels of *lde* were increased in four ciprofloxacin-induced strains; however, no significant changes in expression levels of *mdrL* were observed in any of ciprofloxacin-induced mutants. These observations suggested that efflux pump Lde instead of MdrL might be associated with the development resistance to ciprofloxacin in *L. monocytogenes*.

In order to further confirm our assumption, the gene deletion mutants HL06CIP4Δ*lde* and HL06CIP4Δ*mdrL* were constructed in this study. Decreased MIC of ciprofloxacin was observed only in HL06CIP4Δ*lde* compared to that of the parental strain HL06CIP4, suggesting the important role of Lde in the development of ciprofloxacin resistance in *L. monocytogenes*. The complementation of HL06CIP4Δ*lde* restored the ciprofloxacin MIC of the deletion mutant strain to the parental level, confirming that reduced resistance to ciprofloxacin of HL06CIP4Δ*lde* was specifically due to the deletion of *lde*. Notably, a two-fold decrease in ciprofloxacin MIC was observed in HL06CIP4Δ*lde* after the addition of reserpine, indicating that other efflux pumps which extruded ciprofloxacin may be active in this strain and were affected by reserpine.

Ciprofloxacin accumulation in HL06CIP4Δ*lde* was also determined. Ciprofloxacin uptake appeared to be obviously increased in HL06CIP4Δ*lde* compared to that in HL06CIP4, suggesting that the absence of Lde in HL06CIP4 resulted in a great reduction in the efflux activity of ciprofloxacin. As a consequence of adding reserpine, the accumulation of ciprofloxacin in HL06CIP4Δ*lde* was increased and the final amount was nearly identical to that in HL06CIP4, which was consistent with the MIC results.

Ethidium bromide had been found to be the substrate for many efflux pumps in Gram-negative and Gram-positive bacteria (Nikaido, 2005; Schindler and Kaatz, 2016). Results from efflux inhibition testing showed that MICs of EtBr decreased two- to four-fold in four ciprofloxacin-induced mutants after the addition of reserpine, supporting that resistance to EtBr was associated with efflux in these *L. monocytogenes* strains. Godreuil et al. (2003) reported that efflux pump Lde could be involved in the level of susceptibility to EtBr in *L. monocytogenes*. In the current study, the gene deletion mutant HL06CIP4Δ*lde* showed two-fold decrease in the MICs of EtBr, accumulated more EtBr, and exhibited lower efflux activity for EtBr, compared to the parental strain HL06CIP4. These results suggest that EtBr is the substrate for efflux pump Lde and that the absence of Lde decreases the efflux of EtBr. It is believed that both ciprofloxacin and EtBr are efflux substrates for Lde, which could provide a reasonable explanation for cross-resistance to EtBr in ciprofloxacin-induced strains. In the presence of ciprofloxacin, expression of *lde* is upregulated in *L. monocytogenes*, leading to higher efflux activity for ciprofloxacin and EtBr and increased MICs against these antimicrobial agents.

Until now, efflux pump has been recognized as the main mechanism for BC resistance in *L. monocytogenes* (Aase et al., 2000; Müller et al., 2013; Kovacevic et al., 2015). Our results showed that the presence of reserpine reduced MICs of BC for all the ciprofloxacin-induced mutants from 6 to 2 μg/ml, indicating that reserpine could inhibit the efflux activity for BC and result in the increased sensitivity to BC in these strains. Lack of *lde* had an effect neither on MIC of BC in HL06CIP4 nor on its growth in the presence of BC, suggesting that efflux pump Lde is not associated with BC resistance in *L. monocytogenes*. Therefore, increased expression levels of *lde* in ciprofloxacin-induced strains did not result in the increased MICs of BC for these strains in our study. Our observations suggest that cross-resistance to BC in ciprofloxacin-induced strains of *L. moncytogenes* as reported previously (Rakic-Martinez et al., 2011) and demonstrated in this study could be due to other yet unknown mechanisms instead of Lde.

Our results demonstrated that efflux pump Lde plays a significant role in the development of ciprofloxacin resistance in *L. monocytogenes*. As one of the most prescribed broad spectrum antimicrobials, fluoroquinolones prevent ligation reactions of gyrase and topoisomerase resulting in DNA double-strand breaks in bacteria (Redgrave et al., 2014). Repairing of DNA double-strand breaks could induce the SOS response which helps bacteria survive sudden increase in DNA damage (Dörr et al., 2009). In other words, fluoroquinolones are an inducer of the SOS response. It has been reported that expression of *qnrB*, one of the quinolone-resistance determinants, is regulated through LexA, the central regulator of the SOS response (Da Re et al., 2009). Our previous study found that expression of *lde* could be induced by ciprofloxacin (Jiang et al., 2012). The question is whether the regulation of *lde* expression is dependent on LexA. In the current study, this issue was investigated. Our results showed that the deletion of *lexA* had no effect on the expression levels of *lde* in HL06CIP4 in the absence or presence of ciprofloxacin, suggesting that the expression of *lde* is independent of LexA regulation. Further studies should be performed to clarify the regulation of efflux pump Lde.

## Conclusion

In summary, ciprofloxacin sensitive strain of *L. monocytogenes* exhibited decreased susceptibility to this drug after the induction of ciprofloxacin. Cross-resistance to EtBr was observed in ciprofloxacin-induced strains. However, cross-resistance to BC did not occur in our study. Efflux pump Lde played an important role in the development of ciprofloxacin and EtBr resistance in *L. monocytogenes*. Our results suggest that high expression levels of *lde* might contribute to the cross-resistance between ciprofloxacin and EtBr in *L. monocytogenes*. Ciprofloxacin was an inducer for expression of *lde*. Our results also showed that LexA was not involved in the regulation of efflux pump Lde in *L. monocytogenes*.

## Author Contributions

TY, WG, and LS designed and supervised the study. XJ, PX, XX, and SJ performed the experiments. XJ analyzed the data. XJ and TY drafted the manuscript.

## Conflict of Interest Statement

The authors declare that the research was conducted in the absence of any commercial or financial relationships that could be construed as a potential conflict of interest.
